# Geometry-controlled engineering of the low-temperature proximity effect in normal metal–superconductor junctions

**DOI:** 10.3762/bjnano.16.155

**Published:** 2025-12-12

**Authors:** Munisa A Tomayeva, Vyacheslav D Neverov, Andrey V Krasavin, Alexei Vagov, Mihail D Croitoru

**Affiliations:** 1 National Research Nuclear University MEPhI, Moscow 115409, Russian Federation; 2 HSE University, Moscow 101000, Russian Federation; 3 Moscow Institute of Physics and Technology, 141700 Dolgoprudny, Russian Federation; 4 Departamento de Física, Universidade Federal de Pernambuco, 50740-560,Recife-PE, Brazil

**Keywords:** Bogoliubov–de Gennes equations, normal metal–superconductor junction, order parameter, proximity effect, superconductivity

## Abstract

In the ballistic regime at finite temperatures, the proximity effect diminishes following an exponential pattern; however, at low or zero temperatures, this transition alters to a decay characterized by a power law with a dimensionality-dependent exponent. Here, we extend the current understanding of the proximity effect by exploring the role of normal metal–superconductor (NS) junction geometry in altering the spatial propagation of the superconducting order. Specifically, we demonstrate that geometric factors, such as interface curvature, significantly affect the decay exponent of the Cooper pair wave function, with negative curvature increasing the proximity range exponent and positive curvature shortening it. Furthermore, we discuss how the geometry of the NS interface governs the transparency of the clean NS junction and thus influences the proximity effect. These results deepen our understanding of how geometry and the proximity effect interact, which is important for the design and optimization of superconducting hybrid devices.

## Introduction

When a superconductor (SC) is brought into contact with a normal metal (NM) or a ferromagnet (FM), Cooper pairs penetrate the adjacent material, imparting superconducting properties to it. This phenomenon, known as the proximity effect, enables normal material to support supercurrents and to exhibit a reduced density of states near the Fermi level, where a gap opens in the single-particle spectrum as electrons form into Cooper pairs [1–4].

At the same time, unpaired electrons from the normal side scatter into the superconductor, suppressing the superconducting order parameter near the interface [5–7]. In the normal region, the absence of intrinsic attractive electron–electron interaction causes Cooper pairs to break up beyond a characteristic length scale, namely, the normal-metal coherence length, 
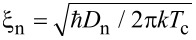
, where *D*_n_ is the electron diffusion coefficient and *T*_c_ is the critical temperature [8–9]. In a FM, the exchange field *E*_ex_ further suppresses superconducting correlations, resulting in a shorter coherence length, 
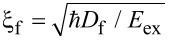
[10–13].

This gradual decay of superconducting correlations in the NM is a hallmark of the proximity effect [14–16]. The pair correlations continuously decrease from their bulk value deep inside the superconductor, leak into the normal material, and eventually vanish at a distance much larger than ξ_n_ far inside the NM [7,17–18]. The spatial dependence of the superconducting pair correlations is characterized by the pair amplitude *F*(*z*), which varies on both sides of the interface [7–8].

The proximity effect in normal metal–superconductor (NS) junctions has been thoroughly studied through experimental [19–21] and theoretical works [7,22–24], which include both pristine and disordered systems [25–26] across a range of temperatures from near absolute zero to higher finite temperatures [27]. A key aspect is the spatial variation of *F*(*z*) at the NS interface and its decay within the normal metal [7].

At temperatures near *T*_c_, in the ballistic regime, the pair amplitude decays exponentially in a NM according to the expression


[1] F(z)∝exp(−K|z|),

where the characteristic decay length is given by 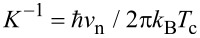 in clean metals and *K*^−1^ = ξ_n_ in dirty metals. Here, *v*_n_ is the Fermi velocity in the NM, and *z* is the distance from the NS interface [2,7,26–28].

However, at low or zero temperatures, self-consistent Bogoliubov–de Gennes calculations show that the decay is no longer exponential. In the ballistic regime, the pair amplitude instead follows a power-law decay [7,29–32],


[2] $$F\left(z\right)\propto {\left(\frac{\xi }{\left|z\right|}\right)}^{\alpha },$$

where ξ is the proximity length. The exponent α depends on the spatial dimensionality of the system [33], taking values of α = 1 in 3D [7,29,32], α = 1/2 in 2D [34–35], and α = 0 in 1D [36]. This behavior holds for distances *z* smaller than both the thermal decay length 
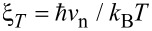
 and the mean free path *l* = *v*_n_τ, where τ is the impurity scattering time [32]. Beyond these scales, the proximity effect is determined by the shorter of these two length scales [37–39]. These results have been obtained for materials and samples with strictly defined dimensionality.

In systems with quasi-low dimensionality or multiband materials, where the single-particle density of states departs from simple integer-dimensional behavior, the power-law decay exponent α can continuously vary between values typical for 3D and 1D systems. This transition reflects how the size and shape of the Cooper pair adapt to the dimensionality of the system [33]. The effective dimensionality of the proximity effect can also be influenced by the geometry of the NS interface. As first shown in [40], the curvature of the interface can significantly affect the proximity effect, that is, a negative curvature (concave, viewed from the SC) enhances the proximity range, while a positive curvature (convex) suppresses it.

Another critical factor is the interface transparency. A perfectly reflective interface (zero transparency) completely decouples SC and NM, suppressing proximity-induced correlations and producing an abrupt change in the Cooper pair density [7,27,41]. Conversely, a perfectly transparent interface yields a continuous order parameter profile across the interface. In realistic systems, finite reflectivity due to band mismatch leads to partial suppression of Andreev reflection and reduced proximity strength [6,42]. Even small interface imperfections can significantly impact superconducting hybrid devices by enhancing normal quasiparticle scattering at the expense of Cooper pair transport [42].

Although interface transparency can be tuned by chemical surface treatments or in situ growth [43], an alternative and less explored approach is to control the proximity effect via the geometry of the NS junction. In this work, we systematically investigate how geometrical characteristics of the NS interface, such as local curvature and morphology, affect both the spatial decay of the superconducting order parameter in the NM and the effective interface transparency. We aim to elucidate how geometric variations modify the amplitude and spatial profile of the Cooper pair wave function, as well as the effective barrier potential at the interface. These geometric effects influence the balance between Andreev reflection and quasiparticle scattering, modulate pair-breaking mechanisms, and thus control the proximity effect. This geometric degree of freedom provides a novel route for engineering and optimizing the performance of superconducting hybrid devices.

## Results and Discussion

### Model

We perform the calculations on a system described by the two-dimensional Hubbard model, defined through the following lattice Hamiltonian [44]:


[3]

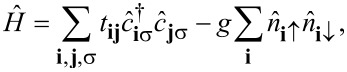



with 
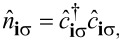
 where 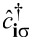 (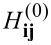) represent electron creation (annihilation) operators for spin σ at site **i** on the lattice. The tunneling amplitude *t***_ij_** is non-zero only between nearest neighbors (*t***_ij_** = −*t*), *g >* 0 is the superconducting pairing constant on the superconducting side of the heterojunction, and *g* = 0 in the normal region [45].

The effective mean-field Hamiltonian associated with Equation 3 is written as [44,46]:


[4]

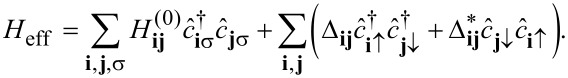



Here 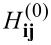 is the single-particle Hamiltonian,


[5] $$H_{ij}^{\left(0\right)}=t_{ij}+\left(V_{i}+U_{i}\right)\delta_{ij}$$

with δ**_ij_** as the Kronecker delta and *U***_i_** as the Hartree potential.

The eigenstates and eigenvalues of *H*_eff_ can be obtained by solving the Bogoliubov–de Gennes matrix equations [5,47–49]:


[6]

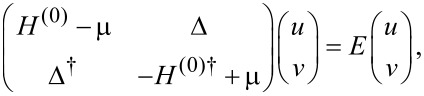



where *u* and *v* are eigenvectors, and *E* the corresponding eigenvalues, μ represents the chemical potential of the system, which is adjusted to have the electron density below half-filling, 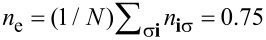, where *N* is the number of lattice sites, to avoid being in resonance with the peak in the single-particle density of states at *n*_e_ = 1.

The order parameter Δ**_ij_** = Δ**_i_**δ**_ij_** and the Hartree potential *U***_i_** are determined from the self-consistency equations. For the order parameter, we have [50]


[7]

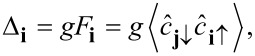



where *F_i_* is a pair amplitude, and for the Hartree potential


[8] $$U_{i}=-\frac{g}{2}\sum_{\sigma }\langle {\hat{c}}_{i\sigma }^{†}{\hat{c}}_{i\sigma }\rangle ,$$

with ⟨…⟩ denoting the quantum mechanical averaging.

The Equations Equation 6 are solved numerically through a self-consistent iteration process that produces the eigenvectors *u* and *v*. These eigenvectors are then used to calculate updated values of the order parameter and Hartree potential, and the process is repeated until convergence is reached at each site [51–53]. In the following, all energy values are given in terms of the hopping amplitude *t*, and all distances in terms of the lattice constant.

**Figure 1:**
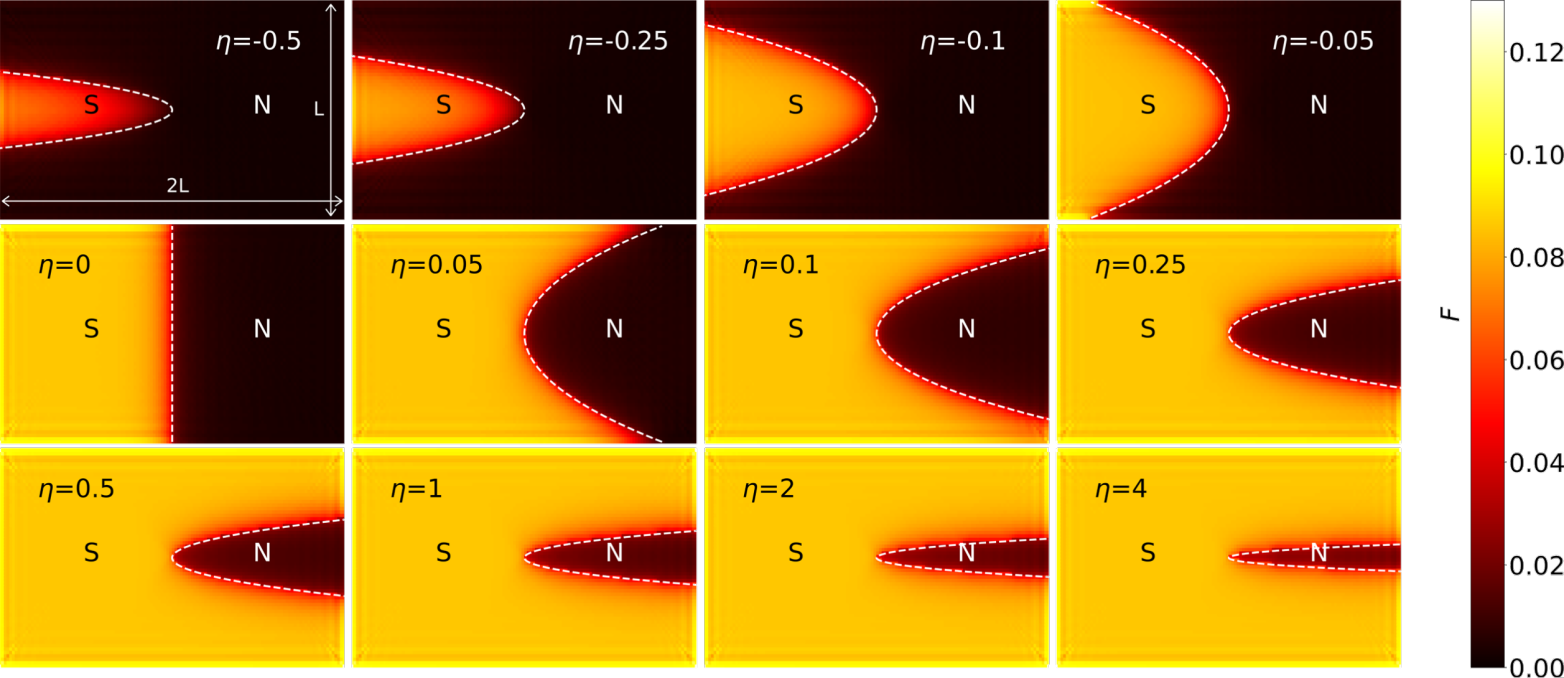
Superconducting correlations in a superconductive sample (S) with normal (N) region of a parabolic shape as a function of the coefficient η characterizing the parabola curvature, *z* = η*x*^2^. The white dashed lines show the interface between the superconductor and the normal metal. The dimensions of the sample are 2*L* × *L* with *L* = 64.

To investigate the influence of NS junction geometry on the superconducting proximity effects, we consider a 2D interface between NM and SC given by a parabolic line in *x*–*z* plane,


[9] $$z=\eta x^{2},$$

where the parameter η can be interpreted as the curvature of the interface at *x* = 0. By varying η, we explore how the curvature affects the spatial decay of superconducting correlations in NM. In the limiting case of η = 0, the NS interface is flat, representing a standard planar junction. For large positive values η ≫ 1, the geometry approaches a quasi-1D NM channel embedded in a superconducting background, effectively forming a “normal wire in a superconducting sea” [54]. In contrast, for η ≪ −1, the system represents a quasi-1D superconducting wire (which can only exist for moderate |η| due to fluctuations) immersed in the NM, essentially a “superconducting wire in a normal sea” [55–56]. Representative configurations for different η values used in subsequent calculations are schematically illustrated by white dashed lines in Figure 1.

For numerical simulations, we used a discretized lattice model with a system size of 128 unit cells along the *z*-direction (the principal axis of the NS transition) and 64 unit cells in the perpendicular *x*-direction. A total of twelve different geometries were modeled by varying η in the set η ∈ {0, ±0.05, ±0.1, ±0.25, ±0.5, 1, 2, 4}.

Figure 1 illustrates the spatial distribution of the superconducting pair amplitude across the NS interface for several selected interface geometries with both negative and positive values of η. Consistent with previous studies [33], the spatial decay of superconducting correlations in the normal and superconducting regions, corresponding to the proximity and antiproximity effects, respectively, exhibits distinct qualitative behaviors. Moreover, both effects are found to be strongly influenced by the interface curvature η, particularly by its sign. For instance, in the normal region, the spatial correlations decay more rapidly when η *<* 0. In what follows, we present a detailed quantitative analysis of how the decay rate varies as a function of η.

**Figure 2:**
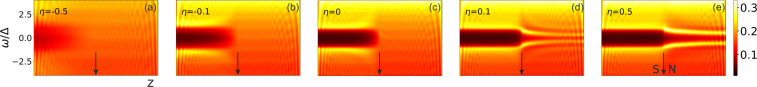
Local density of states normalized to the value of Δ_bulk_ along the symmetry axis. (a) η = −0.5; (b) η = −0.1; (c) η = 0; (d) η = 0.1; (e) η = 0.5. The black arrows show the location of the S–N interface on the *z*-axis.

The proximity effect is further quantified in Figure 2, which illustrates the suppression of the density of states (DOS) at low energies in the NM, that is, the proximity gap, induced by its proximity to the SC. The magnitude of the gap depends on the curvature parameter η. The proximity gap sets the lowest quasiparticle excitation energy scale in the NM region, crucial for coherence and stability of superconducting hybrid devices. For NS junctions with large positive curvature, that is, for quasi-low-dimensional structures, a robust proximity gap emerges, persisting over extended distances because of enhanced superconducting correlations. However, real systems often exhibit a soft gap in experiments, where the DOS remains nonzero at low energies. This broadening arises from inelastic scattering, interface imperfections, and finite quasiparticle lifetimes [46,53,57]. Experimentally, the proximity gap can be probed by tunneling spectroscopy by measuring the differential conductance d*I*/d*V* on the NM side.

### Power-law decay of the pair amplitude

To quantify the decay of superconducting correlations in the normal region, we analyze the spatial profile of the pair amplitude *F*(*z*) along the symmetry axis (*z*-direction). The results show that, as η increases, the superconducting region progressively envelopes the NM region, strengthening the proximity effect. In particular, for large positive values of η, the amplitude of the pair penetrates deeper into the NM, indicating significantly increased superconducting correlations in this region [33,36].

To extract quantitative information about the decay behavior of superconducting correlations into the NM, we fit the computed pair amplitude profiles to a generalized power-law decay function of the form


**Figure 3:**
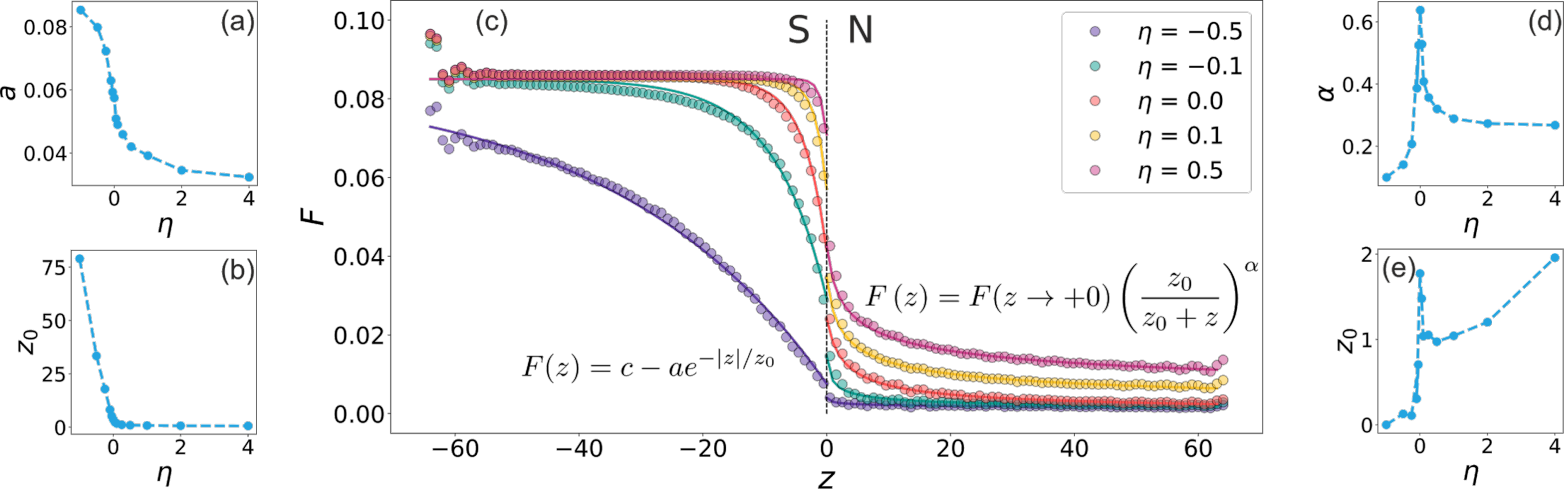
Spatial distribution of the superconducting pair amplitude across the NS junction. (a, b) Fitting coefficients *a*, *z*_0_ as functions of the curvature η for the functional dependence of the superconducting pair amplitude 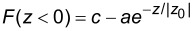 in the superconducting region. (c) The value of the superconducting pair amplitude along the *z*-axis at *x* = 0; circles represent numerical data, and solid lines correspond to fitted dependencies. (d, e) Fit coefficients α, *z*_0_ as functions of the curvature η for the functional dependence of the superconducting pair amplitude *F*(*z >* 0) = *F*(*z*→+0)[(*z*_0_)/(*z*_0_ + *z*)]^α^ in the normal region.

[10] $$F\left(z>0\right)=F\left(z\to +0\right){\left(\frac{z_{0}}{z_{0}+z}\right)}^{\alpha },$$

where *z*_0_ is a characteristic length scale (proximity length, related to superconducting length [32]), *F*(*z*→+0) is the numerical value of the pair amplitude in the vicinity of the interface, and α is the power-law decay exponent. Representative fits of the numerical data to this functional form are shown by the solid lines in Figure 3c. The fitting results for all values of η are summarized in Figure 3d,e, where the extracted parameters *z*_0_ and α are plotted as functions of η. From Figure 3e, we observe that *z*_0_ increases with increasing η. This implies that the proximity length grows as the geometry becomes more confining for the NM (i.e., as the system approaches a quasi-1D “normal wire embedded in a superconductor”). This is consistent with the physical picture that the Cooper pair wavefunction becomes more spatially squeezed in the normal region [33,58].

Figure 3d is particularly informative, showing how the power-law decay exponent α varies with η. For negative values of η (i.e., when the superconductor forms a quasi-1D wire), α increases, indicating a faster decay of the pair amplitude in the normal region. For positive η, the exponent decreases, corresponding to slower decay and enhanced superconducting correlations. Interestingly, at η = 0, the pair amplitude reaches a maximum value of approximately α(η = 0) ≈ 0.6, consistent with previous studies of clean two-dimensional systems, where values near 0.5 were reported [33–35]. As η increases, α(η) decays and asymptotically approaches the typical values of effectively one-dimensional systems, as expected [36].

### Exponential recovery of the pair amplitude in the inverse proximity effect

In a SC, superconductivity is intrinsic. The suppression of the order parameter, that is, the inverse proximity effect, is a localized response to the boundary condition or interface, and the pairing potential Δ_s_(*z*) must recover to its self-consistent bulk value Δ_bulk_ on a characteristic length scale set by the superconducting coherence length ξ_s_[5,59–60].

In the SC, near the NS boundary, the superconducting order parameter Δ_s_(*z*) is governed by the self-consistent Bogoliubov–de Gennes equations. Linearizing these equations under the assumption of a weak perturbation (i.e., Δ_s_(*z*) ≈ Δ_bulk_), one finds that


[11] $$\Delta_{\text{s}}\left(z\right)\approx \Delta_{\text{bulk}}\left[1-Ae^{-z/\xi_{\text{s}}}\right],$$

where *A* is a constant determined by interface transparency (γ_NS_) and material mismatch (γ). This exponential recovery arises from the mean-field self-consistency in the BCS theory and the gapped quasiparticle spectrum of the superconductor. The system energetically favors a homogeneous pairing amplitude, and any deviation from it decays on a characteristic scale ξ_s_, as subgap quasiparticles cannot propagate far into the bulk. Consequently, even at zero temperature, the antiproximity effect is a short-range phenomenon, in contrast to the long-range power-law decay of proximity-induced pairing in the normal metal.

We quantitatively investigate how the curvature of the NS interface modifies this behavior by analyzing the spatial profile of the pair amplitude *F*_s_(*z*) inside the superconducting region near the interface for various values of η. The results shown in Figure 3c for *z <* 0 reveal that in certain geometrical configurations, particularly for highly negative values of η, where a narrow superconducting channel is surrounded by a normal metal, the suppression of superconducting order can be substantial. To quantify this dependence, we fit the numerical results in the phenomenological form:


[12] $$F\left(z\right)=c-ae^{-z/\left|z_{0}\right|}.$$

The parameter *c* is almost curvature-independent, and its value is *c* ≈ *F*_S,bulk_. Other fit results are shown in Figure 3a,b. The parameter *z*_0_ is a decay length that decreases with η: For positive curvature, *z*_0_ remains small and constant, while for negative curvature, it increases monotonically with |η|. We also observe that the parameter *a*, which mimics *A*, exhibits a strong curvature-dependence, reflecting changes in the transparency of the interface and in the local density of states near the interface.

**Figure 4:**
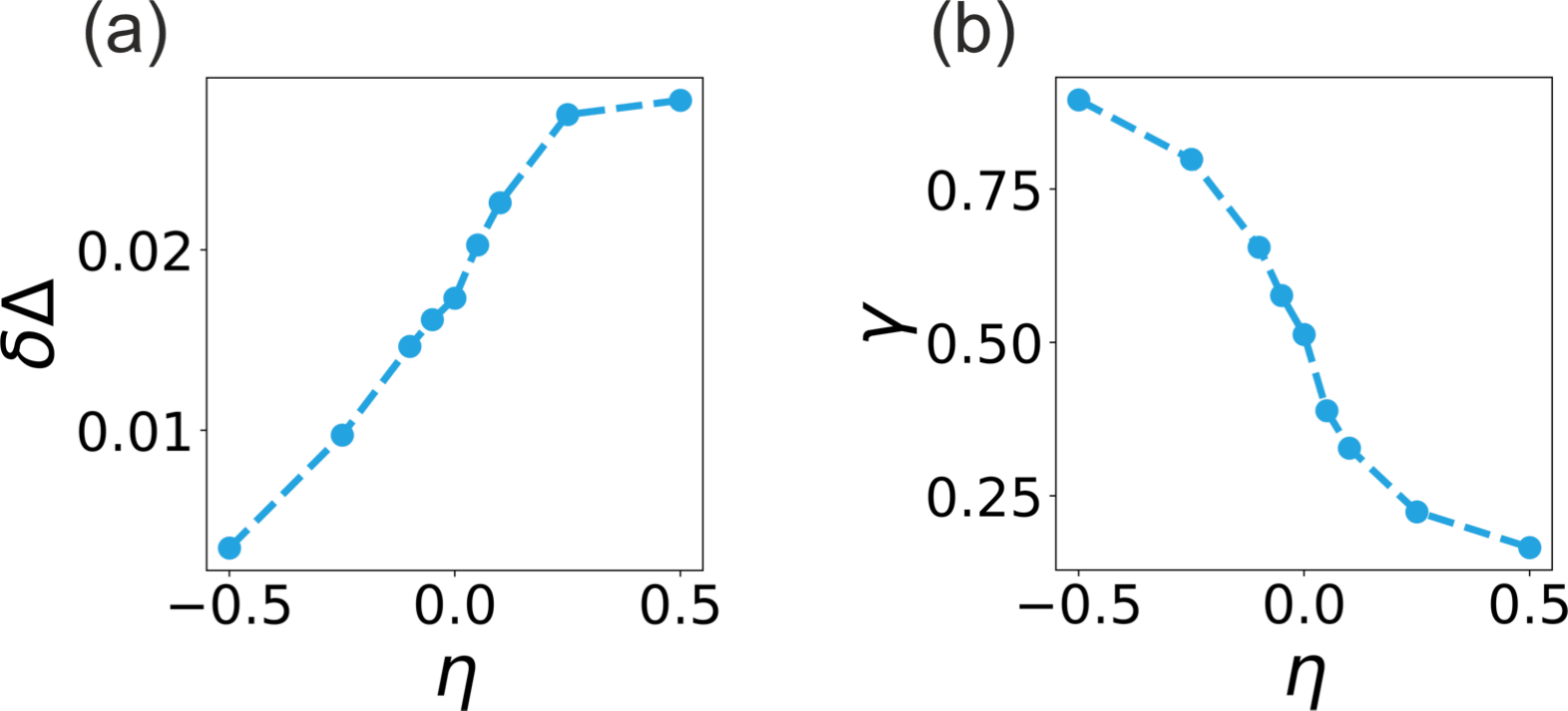
(a) The difference in gaps at the NS junction as a function of the NS junction curvature parameter. (b) The interface pair-breaking parameter as a function of the NS junction curvature parameter.

We quantify this suppression of the order parameter/pair function amplitude in the SC due to the proximity effect in terms of a pair-breaking parameter,


[13] $$\gamma =1-\frac{F\left(z\to -0\right)}{F_{\text{S},\text{bulk}}},$$

which effectively describes how the boundary to the normal metal acts as a source of pair breaking. Figure 4b illustrates the dependence of γ on the geometry of the NS junction, as extracted from the numerical calculations of the spatial dependence of the superconducting gap shown in Figure 3c. The results show a monotonic decrease in pair breaking as the geometry changes from η ≪ −1 to η ≫ 1. This trend is expected since, for a “normal wire in a superconducting sea”, the influence of the boundary to NM diminishes.

### Proximity-induced gap and interface transparency

The proximity-induced gap (mini-gap) on the NM side of a NS junction, shown in Figure 3c, is generally smaller than the superconducting gap Δ_s_(0) at the interface. This mismatch or jump between the gaps on both sides of the interface grows at larger value of curvature η. This is shown in Figure 4a, which reveals a monotonically increasing dependence δΔ*(η)*.

The obtained monotonic increase of the mismatch in the gaps is consistent with the earlier results obtained for disordered superconductons described by the Usadel theory, which predicts that the value of the jump depends on the interface resistance. Solving the Usadel equations together with Kupriyanov–Lukichev boundary conditions for a flat interface [61] at *T* = 0, one obtains the following implicit relation for the proximity-induced gap on the NM side [62]:


[14] $$\frac{\Delta_{\text{n}}}{\Delta_{\text{s}}\left(0\right)}={\left[1+\gamma_{\text{NS}}\frac{\beta }{\pi }\sqrt{1-\frac{\Delta_{\text{n}}^{2}}{\Delta_{\text{s}}^{2}\left(0\right)}}\right]}^{-1},$$

where β = Δ_s_(0)/*kT*_c_ = 1.76 is the BCS ratio for a conventional superconductor [63] (*T*_c_ is the critical temperature, Δ_s_(0) is the gap at *T* = 0). The parameter γ_NS_ ≡ σ_n_/(*G*_I_ξ_n_) describes transparency of the interface (its resistance). Here σ_n_ and ξ_n_ denote, respectively, the normal-metal conductivity and coherence length, while *G*_I_ is the interface conductance. Equation 14 shows that increasing γ_NS_ reduces the proximity-induced gap Δ_n_, so the mismatch δΔ = Δ_s_(0) − Δ_n_ between the gap values on the two sides of the interface grows when η_NS_ ∝ 1/*G*_I_ increases.

The numerical results for the pair amplitude *F* in Figure 3c are consistent with this prediction if one takes into account that *G*_I_ is proportional to the transmission probability *T* across the interface. This quantity depends on both the transmission probability of the individual conduction channels near the Fermi surface and on the total number of such channels. Both contributions are expected to decrease as the curvature η grows. At large η, the local width of the normal region near the interface decreases, reducing the number of available transmission modes in the NM due to transverse confinement [64–67] and, thereby, decreasing the number of available transmission channels. At the same time, the coupling between these confined modes on the NM side and the continuum modes on the SC side weakens because of increasing momentum mismatch, which suppresses the transmission probability of the channels through the interface. Together, the reduction in channel number and the suppressed coupling decrease the interface conductance *G*_I_ [68–69]. A lower *G*_I_ corresponds to a larger effective interface transparency parameter γ_NS_, and, according to Equation 14, this results in a smaller proximity gap Δ_n_ and a larger jump δΔ at the interface. This provides a qualitative explanation for the numerical observation that the proximity-induced gap on the NM side diminishes as the curvature η increases.

## Conclusion

In this work, we have systematically investigated the impact of the geometry of a superconductor–normal metal heterojunction on key features of the proximity effect, namely the power-law decay of the Cooper pair amplitude, the effective transparency of the junction, and the induced proximity gap in the normal region. Employing a fully numerical self-consistent solution of the Bogoliubov–de Gennes equations, we analyzed a variety of boundary geometries without relying on simplifying assumptions such as quasiclassical approximations or linearized gap equations. Our approach thus captures both the microscopic structure of the pairing correlations and the influence of boundary-induced inhomogeneities in a unified framework.

We find that the power-law decay of the induced pair amplitude in the normal region is highly sensitive to the shape of the NS interface, as quantified by the exponent α, which varies systematically with the boundary curvature. Likewise, the effective transparency of the interface and the amplitude of the induced proximity gap are strongly modulated by geometric factors. These results demonstrate that the NS boundary geometry appears not merely to be a passive feature of the device but an active design parameter that can significantly alter the strength and spatial extent of superconducting correlations in hybrid structures.

Our findings have important implications for the engineering of superconducting heterostructures, particularly in nanoscale and mesoscopic systems where interface properties can be tailored with high precision. For example, geometric control of the proximity effect may provide an additional degree of freedom for optimizing device performance in superconducting quantum circuits, Josephson junctions, or topological superconducting platforms, where the strength and range of the induced pairing correlations are critical.

Future work could extend our analysis to incorporate the effects of disorder, finite temperature, spin–orbit coupling, and magnetic fields, which are known to interact with geometry in nontrivial ways. Furthermore, comparison with experimental data from hybrid nanostructures with engineered NS boundaries, such as those reported in [40], shows good quantitative agreement with our predictions.

In summary, we have shown that boundary geometry is a key factor in shaping the superconducting proximity effect in NS heterostructures. Our results provide both qualitative insights and quantitative predictions that can guide the design and interpretation of experiments in superconducting hybrid systems.

## Data Availability

Data generated and analyzed during this study is available from the corresponding author upon reasonable request.
